# Application of Atmospheric-Pressure Jet Plasma in the Presence of Acrylic Acid for Joining Polymers without Adhesives

**DOI:** 10.3390/ma16072673

**Published:** 2023-03-28

**Authors:** Roman Günther, Walter Caseri, Christof Brändli

**Affiliations:** 1 Laboratory of Adhesives and Polymer Materials, Institute of Materials and Process Engineering, ZHAW Zurich University of Applied Sciences, 8401 Winterthur, Switzerland; guea@zhaw.ch; 2 Multifunctional Materials, Department of Materials, ETH Zürich, 8093 Zurich, Switzerland; walter.caseri@mat.ethz.ch

**Keywords:** polymer surface modification, nitrogen plasma at atmospheric pressure, polymer surface analysis, reversible bonding, adhesion

## Abstract

This study investigates the treatment of surfaces with jet plasma at atmospheric pressure in the presence of acrylic acid as a resource-saving and efficient approach to joining polymers on polystyrene (PS) and polyamide 12 (PA 12) surfaces. Acrylic acid was added in order to introduce functional groups to the polymer surfaces. XPS analysis revealed a high density of oxygen-containing groups, e.g., carboxylic acid groups, on the polymer surfaces, the detailed composition depending on the polymer. The AFM measurements indicated that the modification of polyamide resulted in morphological changes and an increase in surface roughness due to polymer recrystallization. When the surface-modified polymers were brought in contact under a load, significant adhesion between the polymer surfaces was measured. In particular, PS and PA 12, which are otherwise difficult to join by gluing, could readily be connected in this way. The joint polymers could be separated intentionally by immersion in water, thus enabling the recycling of the materials. The resistance of the joint to water depends on the polymer system, with polyamide providing strikingly higher resistance than polystyrene. Accordingly, treating the joint polymers with water allows debonding on demand, particularly when PS is involved. Exposure of modified polymer surfaces to solutions of metal ions increased the resistance of joint polymers to water.

## 1. Introduction

Plasma treatment at low pressures with gases such as oxygen, nitrogen, helium, hydrogen, argon, and ammonia, or mixtures of the stated gases, is frequently employed in polymers to modify their surfaces. Depending on the type of plasma and the polymer, functional groups, such as hydroxyl, carbonyl, carboxyl, amine, nitro, and peroxide groups, are introduced to the surfaces [1,2,3,4,5,6,7,8,9,10]. Often, such treatments are performed with the aim of improving the performance of adhesives. However, surface modification with oxygen plasma was also employed for joining polymers directly, also called autohesion [11,12], i.e., without an adhesive [13]. The resulting adhesion was strong and even exceeded the strength of the substrates.

Nonetheless, the joint could be easily separated within seconds by exposure to water, thus allowing detachment of the substrates on demand. Further modification of such polymer surfaces with acrylic acid with a wet chemical process increased the density of functional groups. These groups were able to coordinate with copper(II) ions, leading to an increase in adhesion and resistance toward water [14]. Accordingly, the joint polymers could not be detached with the use of water anymore. However, separation could be achieved by exposing the joint polymers to strong complexing agents, such as EDTA (ethylenediaminetetraacetate). However, limits in the reproducibility of this method are mentioned [14], which might be associated with the two-step process (first plasma treatment, followed by a reaction in a solution). In any case, a one-step process would be more straightforward, as well as the use of a jet plasma operating at atmospheric instead of low pressure, as atmospheric-pressure plasmas can be readily scaled up to meet industrial production demands and eliminate the need for a vacuum [15,16,17,18,19,20,21,22].

The corona discharge process in air, which is also referred to as corona treatment, has been utilized to treat polymers with plasmas at atmospheric pressure, as documented in previous studies [23,24,25]. Recently, the emergence of atmospheric-pressure jet plasma has widened the scope for conducting experiments under atmospheric conditions with diverse gases and has further enabled the blending of primary gases with organic compounds to graft functional groups onto polymer surfaces [26,27,28,29,30,31,32].

Accordingly, this study explores the modification of surfaces with a plasma jet at atmospheric pressure and acrylic acid as a supplementary agent. The emphasis was placed on the plasma process, particularly in comparison to the wet chemical modification that has been previously reported [14]. The primary gas chosen for the plasma modification was nitrogen to prevent the complete oxidation of acrylic acid. The impact of the treatment on the adhesion of PS and PA 12 in PS–PS, PA 12–PA 12, and PS–PA 12 joints and the resistance of the joints towards water were investigated. In addition, the influence of metal ions on adhesive properties was explored. The results of this study could pave the way for the development of efficient adhesive systems that facilitate recycling, in some cases, by the detachment of joints upon exposure to water.

## 2. Materials and Methods

### 2.1. Materials

Acrylic acid, manganese (II) acetate tetrahydrate, cobalt (II) acetate tetrahydrate, copper(II) acetate monohydrate, zinc (II) acetate dihydrate (Sigma Aldrich, St. Louis, MO, USA), iron (II) acetate anhydrous, nickel (II) acetate tetrahydrate (VWR International GmbH, Darmstadt, Germany), absolute ethanol (Alcosuisse, Rüti bei Büren, Switzerland), polystyrene (GP 585 X, from Synthos Chemical Innovations, Oswiecim, Poland, M_n_ = 56,079 g/mol, M_w_ = 218,167 g/mol), and polyamide 12 (Grilamid L 16 nat, from EMS-Chemie, Domat-Ems, Switzerland, M_n_ = 30,560 g/mol, M_w_ = 47,110 g/mol) were used as received.

### 2.2. Fabrication of Polymer Substrates

Polymer substrates of 80 mm × 10 mm × 4 mm dimensions were prepared by injection molding, followed by hot pressing, as described before [14]. The PS samples were cooled at room temperature in an upright position, while the PA 12 samples were cooled in liquid nitrogen. The final 1.2 mm thick samples were cut into 7 mm × 7 mm and 20 mm × 20 mm squares using a wire cutter for PS and punched into circular discs with diameters of 8 mm and 16 mm using a punching iron for PA 12.

### 2.3. Plasma Treatment at Atmospheric Pressure in the Presence of Acrylic Acid

An atmospheric jet plasma device (Plasmatreat XYZ400, Plasmatreat GmbH, Steinhagen, Germany) was used for the surface treatment. The plasma generation involved passing nitrogen gas (30 L/min) through a gliding arc discharge at a frequency of 23 kHz and voltage of 280 V. The Plasmatreat XYZ400 device comprised a plasma head (PFW10PAD) and a robot capable of moving the plasma head in the *x*-, *y*-, and *z*-axis, allowing for a uniform and highly reproducible application of plasma to the surface. For the pretreatment step, the substrate was treated with nitrogen plasma at a distance of 10 mm with lines spaced 1 mm apart at a speed of 250 mm/s in three passes. Thereafter, acrylic acid was added to the nitrogen plasma. The acrylic acid was evaporated (30 g/h) by pumping (Mitos P-Pump Advanced, Dolomite Microfluidics, Royston, UK) it into nitrogen at 80 °C and then by introducing the acrylic acid-loaded nitrogen flow (5 L/min) directly into the nozzle of the plasma head. The presence of acrylic acid was indicated by a change in plasma color.

### 2.4. Atomic Force Microscopy (AFM)

Atomic force microscopy (AFM) was conducted as described previously [14]. The surface roughness was calculated by integrating the entire 10 µm × 10 µm scan area. In some images, peaks were trimmed to facilitate a better comparison of the structures.

### 2.5. X-ray Photoelectron Spectroscopy (XPS)

X-ray photoelectron spectroscopy (XPS) was performed as reported previously [14]. In order to correct the charge on the insulating polymer samples, the binding energy scale was calibrated with the adventitious C 1s peak at 285 eV, and the C 1s signals were fitted with the help of a database reference [33]. The information depth was estimated using three times (99.7% of all photoelectrons) the inelastic mean free path calculated by QUASES (QUASES-IMFP-TPP2M, V. 3.0, Odense, Denmark) using a non-relativistic model [34].

### 2.6. Adhesion Tests

For the adhesion tests, a centrifugal adhesion test analyzer (LUMifrac) from LUM GmbH (Berlin, Germany) was employed, as described earlier [13,14]. The polymer substrates were joined together in a hot press at 60 °C for 1 min with a load of 200 kg. After bonding, the joint area (A) was measured manually using a digital measuring microscope (VHX-6000 V3.0.0.116, Keyence International, Mechelen, Belgium) and Photoshop (V24.0.0, Adobe, Dublin, Ireland). The samples were stored at room temperature in a dry atmosphere until they were tested in the LUMifrac device. Up to eight samples were inserted together in the measuring chamber.

### 2.7. Debonding Experiments

In order to investigate the detachment of joint polymer surfaces, a digital microscope (VHX-6000 V3.0.0.116, Keyence International, Mechelen, Belgium) was used. The samples were placed in a Petri dish and kept in position with tweezers (Figure S1). Immediately after filling the Petri dish with water, an optical recording was commenced at a rate of 15 frames per second for 15 min. Thereafter, the frames were recorded at 2 min intervals. The detachment time was determined by the evaluation of the recorded frames.

### 2.8. Metal(II)-Ion Loading

Solutions comprising 0.025 M salts of Mn^II^, Fe^II^, Co^II^, Ni^II^, Cu^II^, and Zn^II^ acetate were prepared in ethanol, resulting in the complete dissolution of the compounds. The polymer surfaces were exposed to a droplet of the respective solutions. After 10 s of exposure, the samples were rinsed with 10 mL of ethanol to remove the excess ions. Finally, the samples were blown dry with air and stored in a dry atmosphere.

## 3. Results and Discussion

### 3.1. Effect of Plasma Modification on Surface Structure and Chemistry

**Figure 1:**
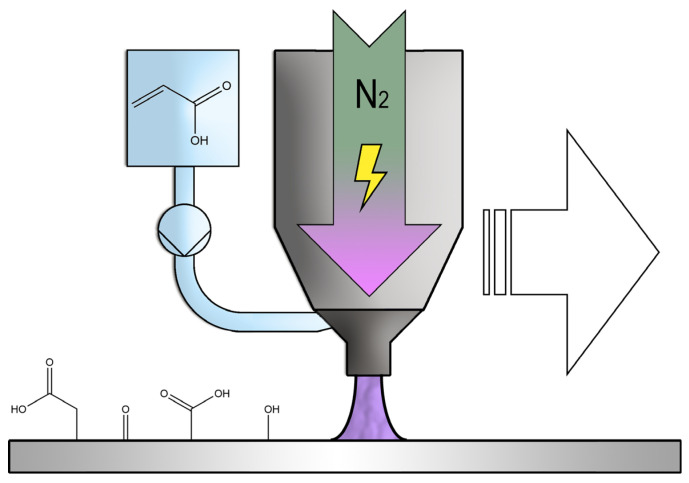
Diagram of the experimental setup for plasma treatment at atmospheric pressure in the presence of acrylic acid.

The PS and PA 12 surfaces were modified using an atmospheric jet plasma method, which generates plasma at ambient pressure through a gliding arc discharge. The plasma is ejected to the surface via a fast gas flow through a nozzle. Acrylic acid was directly introduced into the plasma jet, leading to plasma-assisted deposition onto the polymer surfaces (see Figure 1). A robot passed the plasma jet over the surface multiple times at constant speed and distance.

**Figure 2:**
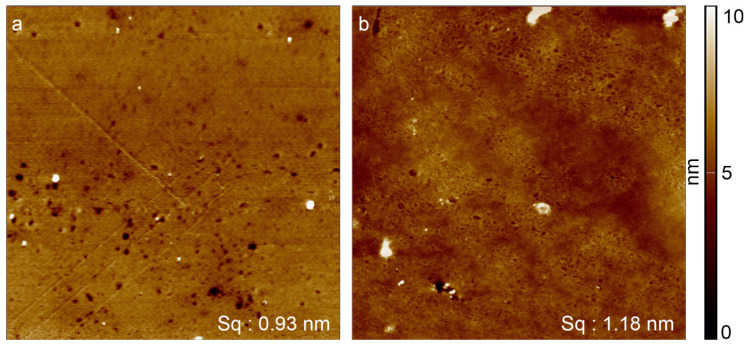
AFM images of PS before and after atmospheric plasma jet treatment. The images have an edge length of 10 µm. (**a**) Unmodified PS surface with a roughness of 0.93 nm (S_q_). (**b**) PS surface after exposure to a plasma containing acrylic acid at atmospheric pressure, with a roughness of 1.18 nm (S_q_).

The AFM images in Figure 2 illustrate the surface of PS before and after the plasma treatment. The roughness of the surfaces was calculated using the underlying 3D data of the AFM measurements. The non-modified samples (Figure 2a), produced by injection molding and hot pressing, had a smooth surface (roughness 0.93 nm), which did not change significantly after the plasma treatment (roughness 1.18 nm) (Figure 2b). Additionally, the morphology of the surface remained essentially the same after the plasma treatment, without aggregates, distortions, or stains. Thus, the products generated by the treatment appear to be thin and evenly distributed since they do not cover the underlying structure of the substrate.

**Figure 3:**
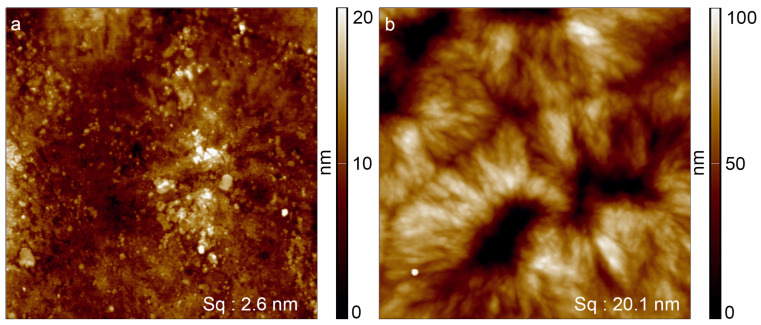
AFM images of PA 12 before and after atmospheric plasma jet treatment. The images have an edge length of 10 µm. (**a**) Unmodified PA 12 surface with a roughness of 2.6 nm (S_q_). (**b**) PA 12 surface after modification with a plasma containing acrylic acid at atmospheric pressure, with a roughness of 20.1 nm (S_q_).

Figure 2 displays the AFM images of PA 12 surfaces. Untreated surfaces of PA 12 (Figure 3a) exhibited higher roughness (2.6 nm) than that of PS (0.93 nm). This might be due to the semi-crystalline nature of PA 12, where semi-crystalline domains could lead to structural features. After the plasma treatment (Figure 3b), a significant increase in roughness of PA 12 (from 2.6 nm to 20.1 nm) was observed, which was attributed to heat-induced recrystallization [35] under the action of the plasma, which resulted in larger and more prominent crystals. However, apart from the recrystallization phenomena, no further changes, such as the agglomeration of acrylic acid, were observed, indicating that the applied acrylic acid layer on PA might also be thin and uniform.

**Figure 4:**
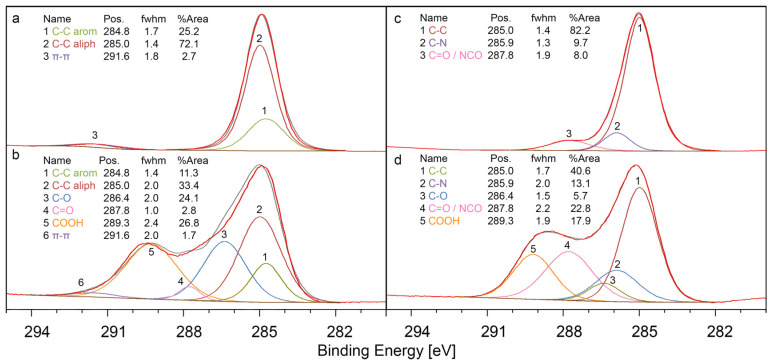
XPS data of the C 1s signals. (**a**) Unmodified PS surface. (**b**) PS surface after exposure to a plasma jet containing acrylic acid. (**c**) Unmodified PA 12 surface. (**d**) PA 12 surface after plasma jet modification with acrylic acid. The spectra were normalized (min to max). C-C arom: aromatic carbon in PS. C-C aliph: aliphatic carbon in PS.

The chemical composition of the surfaces was analyzed with XPS. The high-resolution carbon 1s (C 1s) spectra, as shown in Figure 4, reveal that PS itself, an apolar polymer, does not possess a considerable number of polar functional groups (Figure 4a). However, after the plasma treatment, a significant increase in functional groups was observed on the surface (Figure 4b). The most prominent functional groups were C-O (24.1% of all C atoms) and COOH, which could also indicate ester groups (26.8% of all C atoms), with C=O (2.8% of all C atoms) also being present. The high concentration of COOH suggests the successful deposition of acrylic acid onto the surface. In contrast, PA 12, a polar polymer, exhibited functional groups even before modification (Figure 4c). Specifically, 9.7% of all C atoms were present as C-N links, with an additional 8.0% as NCO or C=O groups, matching the theoretical values of PA 12 (8.3% of all C atoms each). The modification with acrylic acid resulted in a substantial increase in functional groups (Figure 4d), with the proportion of C-N and C=O/NCO groups rising to 13.1% and 22.8% of all C atoms, respectively. Furthermore, 17.9% of all C atoms were present as COOH and 5.7% as C-O groups. Again, the marked increase in COOH attests to the involvement of acrylic acid on the surface.

A comparison between the modified PS and PA 12 surfaces indicates that a greater number of functional groups is present on the PA 12 surface and that the proportion of these functional groups varies depending on the base material. This suggests that functionalization depends on the base material and is not simply a result of the deposition of a layer of products derived from acrylic acid on the surface.

As a side note, the fact that the signal of the π-π* shake-up satellite of PS can be detected before and after modification thus implies that the deposited layer upon modification is thinner than the information depth of XPS, which is approximately 11 nm for PS (calculated with QUASES).

### 3.2. Adhesion of Joints and Resistance towards Water

As PS and PA 12 could be modified with acrylic-acid-containing plasma, the bonding capabilities between the combinations PS–PS, PA 12–PA 12, and PS–PA 12 could be explored. Note that the surfaces of the pristine polymers are incompatible [36,37] due to the apolar nature of the former and the polar nature of the latter.

A significant adhesion was established in all modified cases by bringing the samples in contact under a load. The adhesive strength was determined with a LUMifrac adhesion analyzer [38,39], which measures up to eight specimens in parallel by exerting butt tensile force via an increased rotational speed in the rotating measuring chamber at room temperature. Upon bond failure, a sensor is triggered by the detachment of a copper weight, and the corresponding force is recorded.

**Figure 5:**
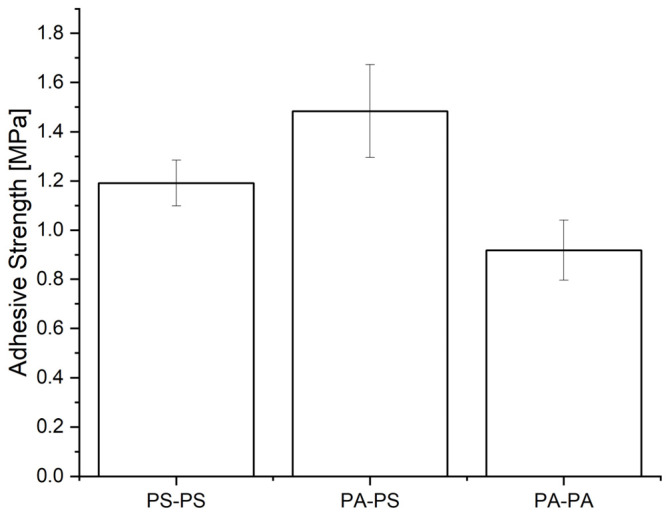
Adhesive strengths were measured with a LUMifrac device for three different material combinations of bonded substrates. PS–PS represents two PS specimens, PA–PS represents a combination of one PS and one PA 12 specimen, and PA–PA represents two PA 12 specimens. Each experiment was performed on four treated samples. The error bars indicate the standard deviation.

Figure 5 displays the strength of the joints, where PS–PS represents the combination of two PS specimens, PA 12–PS is the combination of one PS and one PA specimen, and PA 12–PA 12 represents the combination of two PA 12 specimens all treated with the plasma jet. The difference in adhesive strength of the PA 12–PS system hardly differed statistically from the PS–PS system, indicating successful surface functionalization for both polymers. The adhesive strength of the PA 12–PA 12 system was somewhat lower but still in a similar range.

The resistance of the joint samples to water was investigated by exposure to water in a Petri dish under a microscope. The images were taken in specific intervals. This allowed the evaluation of the detachment by optical observation of the images. The results are presented in Table 1. The table reveals the time required for the joint to detach. Noteworthy, the detachment time depended significantly on the material combinations. For example, the system with two PS specimens (PS–PS) detached after an average of 11 s, while the system with PS and PA 12 specimens (PA 12–PS) took an average of 113 s to detach. The system with two PA 12 surfaces (PA 12–PA 12) showed very high detachment times (>10,000 s).

In conclusion, the strength of the joints was in the same order of magnitude regardless of the substrate combination, while the detachment time varied significantly based on the material pairings. This suggests that the surface groups of plasma-treated PA 12 may form stronger bonds, which are more stable against disruption in water. However, the high roughness of the PA 12 surfaces may also limit the contact area between the substrates and, thus, the number of chemical interactions between the surfaces of the two substrates. Accordingly, when PA 12 is involved, fewer but stronger chemical interactions between the substrates might be established, which leads, coincidentally, to a similar mechanical strength compared to PS, where somewhat weaker but more bonds might be formed. The above-reported XPS measurements imply that the surfaces of PS and PA 12 contain different functional groups. It basically cannot be excluded that bonds of different strengths might be established with regard to the two polymers. However, other explanations might also be consistent with the results, as it still remains unclear which functional groups would cause stronger and weaker bonds between the surfaces.

### 3.3. Effect of Adsorbed Metal Ions on the Joints

In the case of the polymer surfaces modified with acrylic acid via a wet chemical process [14], the adsorption of copper(II) ions enhanced the adhesion strength of the joints and their resistance to water. The underlying mechanism involves, most likely, the formation of coordination bonds of copper(II) ions with oxygen-containing surface groups, in particular, carboxylate groups [14,40]. In order to investigate if these phenomena also occur upon related surface modification by atmospheric-pressure plasma, one side of a polymer, preferably PS, was exposed to a solution of copper(II) ions before contact with another substrate. Subsequently, the XPS measurements indicated the presence of 5.4 atomic percent (At%) copper on PS and 5.2 At% copper on PA 12 surfaces (Figure S2). The detachment times of PS–PS, PA 12–PS, and PA 12–PA 12 and the adhesive strength of PS–PS were assessed.

**Figure 6:**
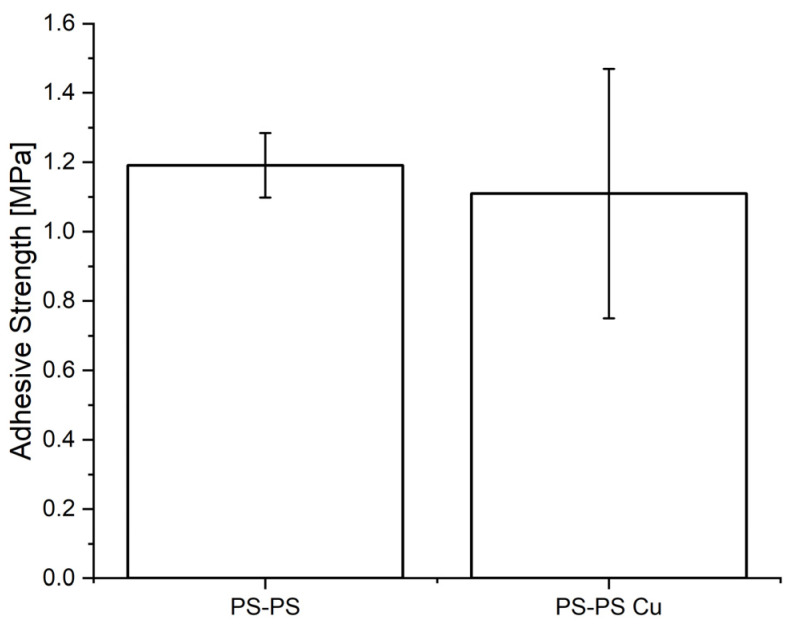
Comparison of adhesive strength measured with a LUMifrac device between PS–PS without and with copper(II) ions adsorbed to the surface before bonding. PS–PS represents a joint of two PS specimens. Each experiment was performed with four samples. The error bars indicate the standard deviation.

Table 2 reveals that the detachment times of the PS–PS and PS–PA 12 substantially increased in the presence of copper(II) (in the case of PA 12–PA 12, the detachment time was on the edge of the observation period also without copper(II) treatment). For instance, the average detachment time increased from 11 s to 358 s for PS–PS and from 113 s to 14,400 s for PA 12–PS. The results presented in Figure 6 suggest that the addition of copper(II) ions did not significantly increase the adhesive strength of PS–PS.

The lack of adhesive strength enhancement by incorporation of copper(II) in contrast to the modification with acrylic acid in the wet chemical process [14] might be attributed to the lower copper(II) loading on the surface than in the related wet chemical process.

In comparison, the specimens grafted with acrylic acid in a wet chemical process yielded 31 At% copper(II) adsorption [14]. Therefore, no further adhesion strength experiments were conducted.

In addition to the copper(II) ions, the impact of manganese(II), iron(II), cobalt(II), nickel(II), and zinc(II) on the joint strength and detachment time was also tested. However, none of these metal ions led to a better performance than what occurred with copper(II), although the detachment time significantly increased upon treatment with any of the metal(II) ions compared to the surfaces without metal ions. This is consistent with the strength of the coordination bonds reported by Irving and Williams [41].

## 4. Conclusions

The treatment of polymers with nitrogen plasma at atmospheric pressure in the presence of acrylic acid is a promising alternative to the related wet chemical methods. This was demonstrated with PS and PA 12, where a high density of functional groups on the surface was generated with this method. The process offers advantages over traditional wet chemical methods, including reduced usage of chemical substances, faster processing times, higher reproducibility, and scalability to large-scale industrial processes. The nature and quantity of the functional groups’ properties depend on the polymer. Moreover, semi-crystalline polymers can exhibit recrystallization effects, which can increase surface roughness. The functional groups at the surface of each polymer allowed the joining of PS and PA 12 with considerable adhesive strength without using adhesives. Importantly, incompatible polymers, such as PS and PA 12, could also readily be joined. The detachment time upon exposure to water strongly depends on the involved polymers, with differences of several orders of magnitude. While the use of metal(II) ions does not appear to impact the strength of the joints, it has a notable effect on detachment times.

## Figures and Tables

**Table 1:** Detachment time of three different material combinations of joint materials. PS–PS represents two PS specimens, PA 12–PS is a combination of one PS and one PA specimen, and PA 12–PA 12 is two PA 12 specimens. Each experiment was performed on two samples.

Material	1st Sample [s]	2nd Sample [s]	Average [s]
PS–PS	18	5	11
PA 12–PS	128	98	113
PA 12–PA 12	>72,000	25,400	-

**Table 2:** Detachment time of three different material combinations of joint substrates with adsorbed copper(II). PS–PS represents two PS substrates, PA 12–PS is a combination of one PS and one PA specimen, and PA 12–PA 12 is two PA 12 specimens. Each experiment was performed on two samples.

Material	1st Sample [s]	2nd Sample [s]	Average [s]
PSPS Cu	196	520	358
PAPS Cu	2800	26,100	14,400
PAPA Cu	>61,000	>61,000	-

## Data Availability

Not applicable.

## Supplementary Materials

The following supporting information can be downloaded at: https://www.mdpi.com/article/10.3390/ma16072673/s1, Figure S1: Setup of the debonding experiment; Figure S2: XPS survey spectra of copper(II) loaded surfaces.
