# Supplementary material for: Application of Atmospheric-Pressure Jet Plasma in the Presence of Acrylic Acid for Joining Polymers without Adhesives
Source: Materials (Basel). 2023 Mar 28;16(7):2673. doi: 10.3390/ma16072673 (PMC10095700; doi:10.3390/ma16072673)
Supplement: Supplementary file 1 [file materials-16-02673-s001.zip › materials-2301183-supplementary.pdf]

Supporting Information

Application of Atmospheric-Pressure Jet Plasma in the Presence of Acrylic Acid for Joining Polymers without Adhesives

Roman Günther [1, 2], Prof. Dr. Walter Remo Caseri [2], Prof. Dr. Christof Brändli[1*]

[1] Laboratory of Adhesives and Polymer Materials, Institute of Materials and Process Engineering, ZHAW Zurich University of Applied Sciences, 8401 Winterthur, Switzerland; guea@zhaw.ch

[2] Multifunctional Materials, Department of Materials, ETH Zürich, 8093 Zurich, Switzerland; walter.caseri@mat.ethz.ch

* Correspondence: christof.braendli@zhaw.ch

2. Materials and Methods

2.7. Debonding Experiments

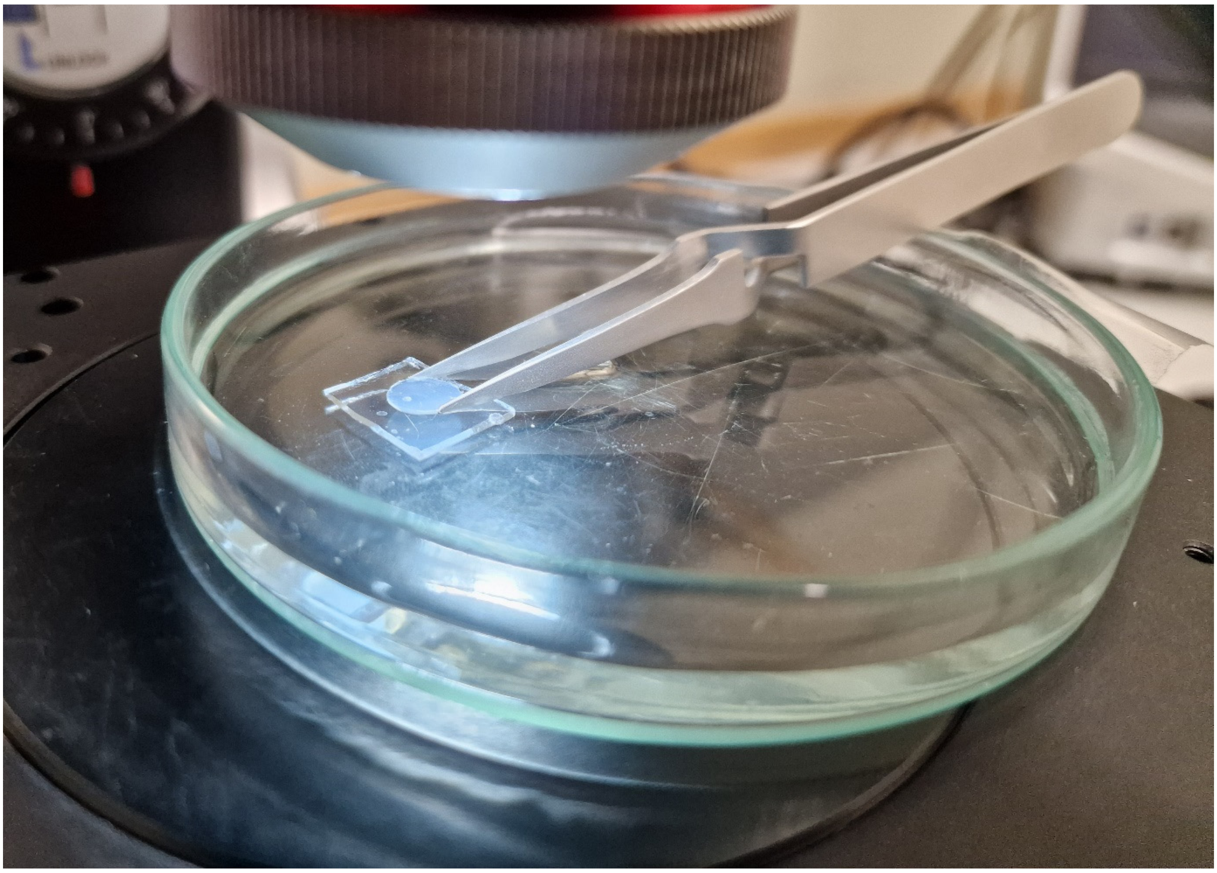

Figure S1. Experimental setup for the debonding experiments.

3. Results and Discussion

3.3. Effect of Adsorbed Metal Ions on the Joints

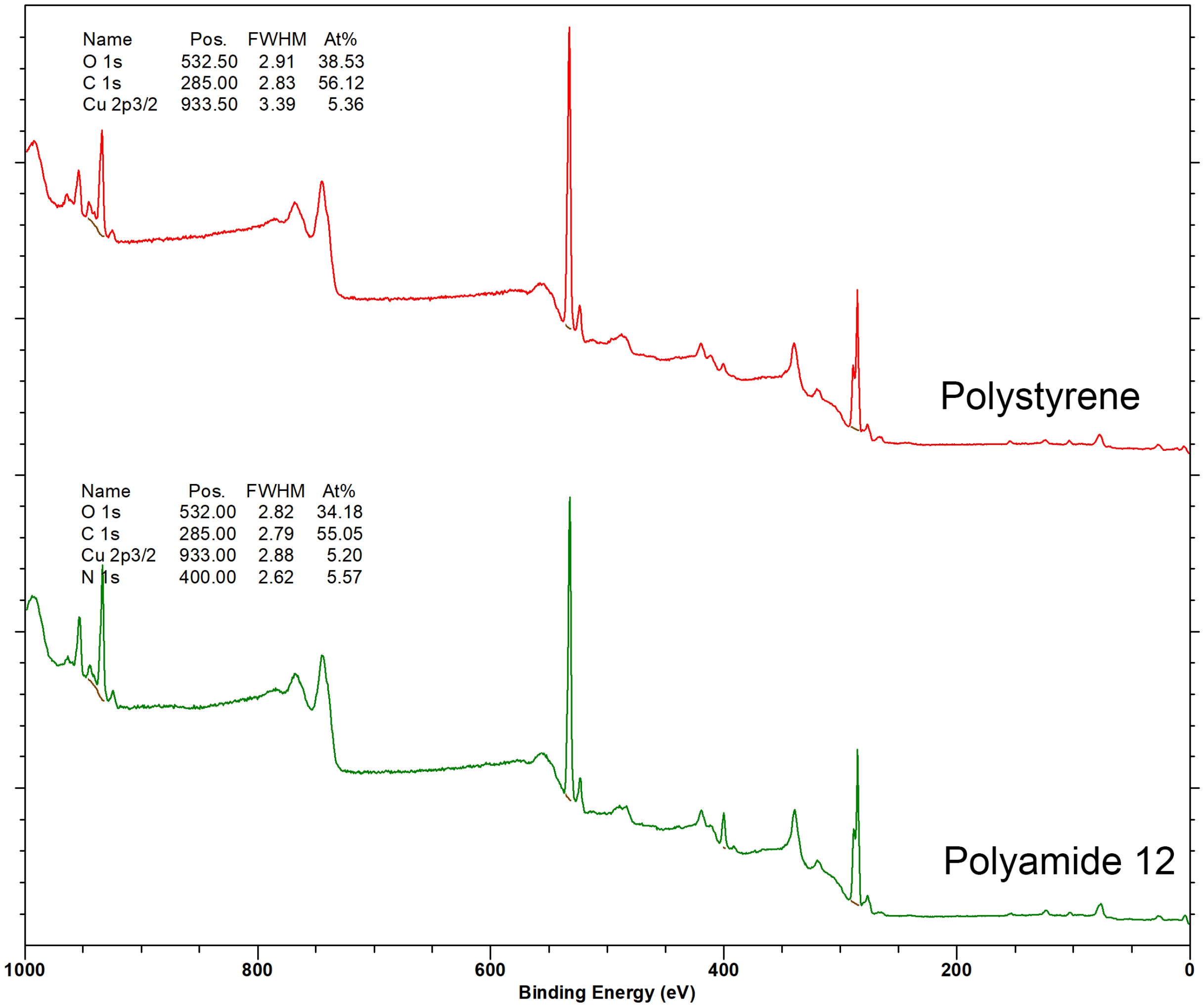

Figure S2. XPS survey spectra, utilized to quantify the copper present on the surfaces of PS and PA 12 after exposures to the copper ion solution.
